# Effect of integrated care model on urinary retention after radical hysterectomy for cervical cancer

**DOI:** 10.12669/pjms.41.4.9917

**Published:** 2025-04

**Authors:** Qiang Zhao

**Affiliations:** 1 Qiang Zhao, Gynaecology and Obstetrics, Tangshan People’s Hospital, Tangshan 063000, Hebei, China

**Keywords:** Cervical cancer, Integrated care model, Radical hysterectomy, Urinary retention, Quality of life

## Abstract

**Objective:**

To explore the impact of integrated care model on urinary retention after extensive hysterectomy for cervical cancer.

**Methods:**

This was a retrospective study. Sixty eight patients with cervical cancer who underwent extensive hysterectomy in Tangshan People’s Hospital from March 2021 to March 2023 were divided into experimental group and control group, 34 cases each. The experimental group received an integrated care model, including periurethral irrigation, pelvic floor muscle and abdominal muscle exercise, induction of urination, bladder function training, and psychological care; the control group adopted a routine care model.

**Results:**

The incidence of complications in the experimental group was lower than that in the control group, and the difference was statistically significant (χ^2^=4.221, P=0.040). After nursing, the SAS, SDS and SF-36 scores of the two groups of patients were significantly improved compared with those before nursing, and the degree of laboratory improvement was higher than that of the control group. The differences were statistically significant (P<0.05). The nursing satisfaction of the experimental group was higher than that of the control group, and the difference was statistically significant (χ^2^=4.660, P=0.031).

**Conclusion:**

Adopting an integrated care model for patients with cervical cancer after extensive hysterectomy can shorten the time of indwelling urinary catheter, reduce residual urine volume and urinary catheter reset rate, improve patients’ bladder function, alleviate patients’ negative emotions, and effectively improve patients’ quality of life.

## INTRODUCTION

Cervical cancer is one of the common gynecological malignant tumors and the fourth most common cancer in women worldwide, seriously threatening women’s health.^1^ Cervical cancer is related to human papillomavirus (HPV) infection, multiple sexual partners, smoking and other factors.^2^ Diagnosis relies on pathological examination. Surgery is the main treatment for early-stage cervical cancer.^3^ Extensive hysterectomy plus bilateral pelvic lymphadenectomy is currently the preferred treatment option for stage IA2 to IB2 cervical cancer.^4^

However, extensive hysterectomy surgery is extensive and invasive, and various postoperative complications are prone to occur. Common postoperative complications include urinary retention, lymphocele, etc., which not only bring physical and mental pain and financial burden to the patient, but are also detrimental to subsequent treatment of cervical cancer.^5^ The integrated care model can further optimize basic nursing measures and provide integrated nursing guidance based on the patient’s individual situation.^6^ This study explored the impact of an integrated care model on urinary retention in patients with cervical cancer after extensive hysterectomy.

## METHODS

This was a retrospective study. Sixty-eight patients with cervical cancer who underwent extensive hysterectomy in Tangshan People’s Hospital from March 2021 to March 2023, and divided into experimental group(n=34) and control group(n=34) according to the random number table method. The experimental group was aged from 36 to 58 years old, with an average age of 42.17 years old, including 16 cases of IA2, 13 cases of IB1, and six cases of IB2. The observation group was aged from 39 to 57 years old, with an average age of 45.32 years old, including 15 cases of IA2 and 114 cases of IB. IB2 5 cases. There was no statistically significant difference in general information such as age and clinical pathological stage between the two groups (P>0.05), and they were comparable.

### Ethical Approval:

The study was approved by the Institutional Ethics Committee of Tangshan People’s Hospital (No.: 2020-019; date: October 15, 2020), and written informed consent was obtained from all participants.

### Inclusion criteria:

- All met the diagnostic criteria for early cervical cancer and confirmed by pathological tissue biopsy.
- According to the International Federation of Gynecology and Obstetrics (FIGO) stage, all are stages IA2 to IB2.
- There are no serious medical diseases.
- The patients voluntarily participated in the study and signed the informed consent form.

### Exclusion criteria:

- Preoperative history of urinary tract infection and bladder dysfunction.
- Combined with other malignant tumors.
- Noble to cooperate with treatment and nursing.

The control group received routine postoperative care: the patients’ vital signs were closely monitored after surgery, symptomatic treatment was provided when symptoms were uncomfortable, and the wards were regularly inspected to observe the condition, drainage volume, and gastrointestinal function.

### Experimental group:

In addition to routine care, a integrated care model was added:

- Paraurethral scrubbing twice a day,
- Instructing patients to perform levator ani, abdominal muscle contraction, and exercise levator ani and abdominal muscles. Consciously tighten and lift the anus, and consciously contract the lower abdomen, lasting 10 seconds each time, 10 times in the morning and evening.
- In the five days after surgery, bladder function training is performed. The urethra is clamped during the day and opened once every 2-4 hours; it is then clamped again after 10 minutes. You can open it at any time if your bladder becomes bloated and you feel the need to urinate.
- Induced urination: Listen to the sound of water when urinating after removing the urinary catheter, and gently massage the lower abdomen.

### Psychological care:

After the operation, medical staff actively communicate with the patients to understand the patients’ understanding and acceptance of the operation. After the operation, they popularize knowledge about postoperative rehabilitation to the patients and their families, so that the patients can understand the key points of postoperative care and related precautions. Carry out psychological counseling to improve the patient’s cognitive level and eliminate the patient’s negative psychological emotions. Explain the possible causes of urinary retention after surgery and how to perform functional training. Let patients and their families understand the occurrence, development and treatment effects of the disease, introduce successful cases in the department, and enhance patients’ confidence in overcoming the disease.

### Outcome Indicators:

- The indwelling urinary catheter time is the time from the day after surgery to the time the patient removes the urinary catheter and can urinate on his own.
- Residual urine volume Use B-ultrasound to measure the residual urine volume in the bladder after complete urination.

Bladder function. Two weeks after the urinary catheter is removed, the patient is instructed to drink water and urinate spontaneously. Abdominal B-ultrasound is used to measure the residual urine volume, and the recovery of the patient’s bladder function is evaluated based on the urine volume. *Grade-I:* The patient’s residual urine volume is less than 50 mL after surgery, and the patient can urinate on his own; *Grade-II:* The patient’s residual urine volume is 50-100 m1, and he has difficulty urinating; *Grade-III:* The patient’s residual urine volume is >100 m1, and he has some difficulty in urinating; *Grade-IV:* The patient has some difficulty in urinating. After removal of the urinary catheter, urination disorder was obvious.

### Urinary catheter replacement rate:

After extubation, the patient cannot urinate smoothly on his own, and the residual urine volume is >100ml, so a new catheter is required to assist urination. The occurrence of complications, the occurrence of fever, urinary tract infection, urinary tract irritation and lymphocele in the two groups were counted

### Psychological status:

Self-rating anxiety scale (SAS) and self-rating depression scale (SDS) were used to compare the psychological status of the two groups of patients before and after care.^7^ The SAS score was 50 points. Those with an SDS score of 53 or above are considered to have depression.

Quality of life Use the Brief Health Questionnaire (SF-36)^8^ to evaluate the patient’s quality of life before and after care, and the score is directly proportional to the quality of life.

Nursing Satisfaction Use the Newcastle Satisfaction with Nursing Scales (NSNS) to conduct patient nursing satisfaction surveys. The full score is 19~95 points, ≥77 points are considered very satisfied, 58~76 points are considered satisfied, and 39 points are considered satisfactory 39~57 is classified as generally satisfied, and ≤38 is classified as dissatisfied. Satisfaction = (very satisfied + satisfied) number of cases/total number of cases × 100%.

### Statistical Analysis:

SPSS 24.0 software was used for analysis. Measurement data were expressed as mean ± standard deviation (*χ̅*±*S*), and t test was used for comparison between groups. Enumeration data were expressed as n (%), and c^2^ test was used for comparison between groups. P<0.05 was considered as a statistically significant difference.

## RESULTS

Patients in the experimental group had shorter post-operative urinary catheter retention time, residual urine volume and urinary catheter replacement rate than the control group. Less than that in the control group, the recovery of bladder function was better than that in the control group, and the differences were statistically significant (P<0.05), Table-I. The incidence of complications in the experimental group was lower than that in the control group, and the difference was statistically significant (χ^2^=4.221, P=0.040), Table-II.

**Table-I:** Comparison of post-operative urinary catheter retention time, residual urine volume, bladder function and urinary catheter replacement rate between the two groups of patients.

Group	Days of Indwelling Urinary Catheter(d)	Residual Urine Volume (ml)	Bladder Function				Replacement Rate of Urinary Catheter
			I	II	III	IV	
Experimental group	14.92±2.47	50.19±3.01	30	2	1	1	4(11.76)
Control group	16.75±4.41	111.62±4.24	20	10	3	1	11(32.35)
t/χ^2^	2.111	68.947	8.333				4.191
P	0.039	<0.001	0.040				0.041

**Table-II:** Comparison of complication rates between the two groups [n(%)].

Group	Fever	Urinary tract infection	Urinary tract irritation sign	Lymphocyst	Total incidence rate
Experimental group	0(0.00)	0(0.00)	1(2.94)	1(2.94)	2(5.88)
Control group	1(2.94)	2(5.88)	3(8.82)	2(5.88)	8(23.52)
χ^2^	4.221				
P	0.040				

Comparison of the SAS, SDS and SF-36 scores of the two groups of patients before care, the difference was not statistically significant (P>0.05); after care, the SAS, SDS and SF-36 scores of the two groups of patients were all the same. It was significantly improved compared with before nursing, and the degree of laboratory improvement was higher than that of the control group, and the differences were statistically significant (P<0.05), Table-III. The experimental group’s nursing satisfaction was higher than that of the control group, and the difference was statistically significant (c^2^=4.660, P=0.031), Table-IV.

**Table-III:** Comparison of psychological status and quality of life between the two groups of patients before and after care (*χ̅*±*S*).

Indicator		Experimental Group	Control Group	t	p
SAS	Pre-intervention	63.71±7.33	63.74±8.88	0.015	0.988
	Post-intervention	41.03±7.13	52.68±9.36	5.772	0.000
SDS	Pre-intervention	65.21±6.68	65.38±8.38	0.096	0.924
	Post-intervention	43.06±7.89	51.50±8.91	4.136	0.000
SF-36	Pre-intervention	51.88±0.81	52.21±0.59	1.884	0.064
	Post-intervention	79.32±2.07	77.53±2.44	3.269	0.002

**Table-IV:** Comparison of nursing satisfaction between the two groups [n(%)].

Group	Very Satisfied	Satisfied	Generally Satisfied	Dissatisfied	Overall Satisfaction
Experimental group	22	9	2	1	31(91.18%)
Control group	16	8	6	4	24(70.59%)
χ^2^					4.660
P					0.031

## DISCUSSION

This study adopts an integrated care model to provide integrated nursing care for various reasons that may lead to urinary retention. Patients in the experimental group adopt an integrated care model. First, psychological care is provided to patients and their families during the perioperative period, and the causes of postoperative urinary retention are explained. Possibly, we can prevent the occurrence of this complication through exercise and preventive measures, enhance the patient’s confidence, better cooperate with treatment, and facilitate the patient’s recovery. Through a series of targeted integrated care, such as paraurethral scrubbing, bladder function exercises, induced urination, etc., we can prevent and reduce the occurrence of urinary retention. The results of this study show that patients under this integrated care model have shorter urinary catheter indwelling time, less residual urine volume, lower urinary catheter reset rate, better bladder function recovery, and lower postoperative complication rates, all statistically significant compared with the observation group. Scientific significance (P<0.05).

Under the integrated care model, through communication with patients and popularization of health knowledge, patients can increase their attention to postoperative rehabilitation, reduce the patient’s stress and anxiety to a certain extent, and encourage them to stay relaxed, improve their negative emotions, and improve their quality of life, and nursing satisfaction are improved, laying a good foundation for postoperative recovery.^9^ The results of this study showed that the improvement in psychological status, quality of life and nursing satisfaction of patients in the experimental group were better than those in the control group, and the difference was statistically significant (P<0.05).Extensive hysterectomy plus bilateral pelvic lymphadenectomy is currently the preferred treatment option for stage IB1 to IB2 cervical cancer.^10^,^11^ Under normal circumstances, when the urine in the bladder reaches a certain level and the intravesical pressure rises enough to stimulate the receptors in the bladder wall, the impulses are transmitted to the sacral spinal cord along the pelvic splanchnic nerves, bladder wall ganglia, etc., causing the parasympathetic urination center in it.^12^,^13^ Excited, urine is excreted from the body. The surgical scope of extensive hysterectomy requires removal of 3-4 cm of the vagina, parametrium, and main sacral ligament. The extensive scope of surgery is the main factor leading to postoperative complications.^14^

Cervical cancer is one of the three major gynecological malignant tumors, seriously endangering women’s health and life safety.^15^ There were 570,000 new cases of cervical cancer in the world in 2018, and 311,000 women died from cervical cancer. This disease seriously threatens women’s health. About 1/5 of new cases of cervical cancer in the world occur in China.^16^ Surveys show that the incidence of cervical cancer in China continues to rise and is getting younger. Surgical treatment is mainly used for patients with early-stage cervical cancer.

The most common postoperative complication is urinary retention. The formation of urinary retention in patients with cervical cancer undergoing extensive hysterectomy is related to the following points:

Due to the wide scope of the operation, 3-4 cm of the vagina, parametrium, and main sacral ligament need to be removed, resulting in the sympathetic nerve fibers, main ligaments, and The pelvic plexus nerves existing in the superficial and deep layers of the sacral ligament, the roots of the pelvic plexus and some extraureteral nerve fibers are inevitably damaged during surgery, which affects the contraction function of the bladder and causes urinary retention.^17^

During the operation, extensive uterine, vaginal and parametrial tissue resection was performed, and the bladder lost its support and became retroflexed.^18^ The posterior urethra and the bladder form an acute angle, and urine accumulates in the bladder and cannot be discharged, leading to urinary retention.

The micturition reflex is suppressed by anesthesia, and the bladder is stretched and squeezed during the operation, resulting in edema and functional impairment.^19^ (4) The influence of the patient’s own psychological condition. Emotions such as tension, anxiety, depression, and fear can lead to aggravation of urinary retention, and the influence of the patient’s own diet and work and rest habits. Urinary retention will not only prolong the patient’s hospitalization time and increase the patient’s financial burden, but also cause psychological burden on the patient, reduce the patient’s quality of life, and affect nursing satisfaction and prognosis.^20^ Therefore, we must attach great importance to the prevention and treatment of urinary retention after cervical cancer surgery, pay attention to patients’ psychological status and quality of life, and improve the quality of care.^21^,^22^

### Limitations:

However, the shortcoming of this study is the small number of patients included.

## CONCLUSIONS

The integrated care model can reduce the postoperative indwelling time of urinary catheters in patients with cervical cancer, reduce residual urine volume and urinary catheter reset rate, help improve patients’ bladder function, alleviate patients’ negative emotions, reduce the risk of complications, and improve their lives. Improved quality and higher satisfaction with patient care.

### Recommendations:

In future clinical work, we will further increase the number of patients in order to more objectively evaluate the impact of integrated nursing intervention on urinary retention in patients with cervical cancer after surgery.
